# Examining nutrition strategies to influence DNA methylation and epigenetic clocks: a systematic review of clinical trials

**DOI:** 10.3389/fragi.2024.1417625

**Published:** 2024-07-15

**Authors:** Isabel García-García, Giorgia Grisotto, Adrian Heini, Simone Gibertoni, Sébastien Nusslé, Semira Gonseth Nusslé, Olga Donica

**Affiliations:** ^1^ Clinique la Prairie, Clarens-Montreux, Vaud, Switzerland; ^2^ Genknowme SA, Lausanne, Switzerland

**Keywords:** nutrition, anti-inflammatory diet, DNA methylation, epigenetic clocks, mediterranean diet (MD)

## Abstract

Nutrition has powerful impacts on our health and longevity. One of the mechanisms by which nutrition might influence our health is by inducing epigenetic modifications, modulating the molecular mechanisms that regulate aging. Observational studies have provided evidence of a relationship between nutrition and differences in DNA methylation. However, these studies are limited in that they might not provide an accurate control of the interactions between different nutrients, or between nutrition and other lifestyle behaviors. Here we systematically reviewed clinical studies examining the impact of nutrition strategies on DNA methylation. We examined clinical studies in community-dwelling adults testing the effects of nutrition interventions on i) global DNA methylation and its proxies, and ii) epigenetic clocks. We included 21 intervention studies that focused on the effects of healthy nutrition patterns, specific foods or nutrients, as well as the effect of multivitamin or multimineral supplements. In four studies on the methylation effects of healthy dietary patterns, as defined by being rich in vegetables, fruits, whole-grains, and nuts and reduced in the intake of added sugars, saturated fat, and alcohol, two of them suggested that a healthy diet, is associated with lower epigenetic age acceleration, one of them reported increases in global DNA methylation, while another one found no diet effects. Studies examining epigenetic effects of specific foods, nutrients, or mixtures of nutrients were scarce. For both folic acid and polyunsaturated fatty acids, the available independent studies produced conflicting findings. Although more evidence is still needed to draw firm conclusions, results begin to suggest that healthy dietary patterns have positive effects on DNA methylation. Additional evidence from large randomized-controlled clinical trials is needed to support the effects of healthy nutrition on the DNA methylome.

## Introduction

Healthy nutrition patterns have powerful effects on general health and, more specifically, on cardio-vascular health (Lichtenstein et al., 2021). In this regard, the Global Burden of Disease has reported that increases in diet quality could help prevent one in five deaths globally (GBD, 2017 Diet Collaborators, 2019). One of the mechanisms that might mediate the effects of nutrition on general health is epigenetics.

Epigenetics comprise the study of genomic changes that do not modify the primary DNA nucleotide sequence. Their mechanisms include changes in methylation, histone modifications, and chromatin remodeling. Epigenetic factors alter gene expression, and in turn, they are modified by environmental factors, such as nutrition, and by internal factors, such as age (Choi and Friso, 2010; Tammen et al., 2013; Pal and Tyler, 2016). DNA methylation is a stable and heritable epigenetic mechanism, and it refers to the addition of a methyl group to a CpG dinucleotide sequence. DNA methyltransferase catalyzes this methylation by using S-adenosylmethionine as the provider of methyl groups. DNA methylation at CpG sites influences gene expression by modifying DNA’s structure, thus affecting the transcription machinery’s access to DNA. This process can either enhance or suppress gene activity by regulating transcription factor binding (Kim and Costello, 2017).

CpG methylation is fundamental during development, since it silences genes whose expression is no longer needed (Pal and Tyler, 2016). With aging, changes in DNA methylation accumulate, and these changes have been associated with genomic instability and with a number of non-communicable disorders, including cardiovascular diseases and cancer (Seale et al., 2022). Aging has been traditionally associated with general decreases in DNA methylation (Lillycrop et al., 2014; Kane and Sinclair, 2019). At the same time, some CpG sites undergo hyper-methylation with age (Horvath, 2013). All these aging-related changes in methylation may be stochastic, meaning that they are random variations in DNA methylation without an apparent cause, or environmentally-related, such as nutrition, stress, and pollution (Lillycrop et al., 2014).

Some of the most common measures of epigenetics in relation with environmental factors, age, and age-related disorders are global methylation and epigenetic methylation clocks. These measures can be largely regarded as complementary. Global DNA methylation provides a comprehensive overview of the methylation state of the epigenome (Li and Tollefsbol, 2021), and it has been associated with vulnerability to diseases such as cancer (Boyne et al., 2018). Environmental factors can modify global DNA methylation, as shown by studies examining the effects of pollutants (Ferrari, Carugno, and Bollati, 2019). Epigenetic methylation clocks are tightly associated with differences in the rates of biological aging (Hannum et al., 2013; Horvath, 2013; Levine et al., 2018). High scores in epigenetic age acceleration are associated with mortality, as well as with the incidence of cancer and cardio-vascular disease (Oblak et al., 2021), while low scores are associated with longer healthspan, or healthy longevity (Jain et al., 2022).

In the specific context of nutrition, previous observational studies have suggested that diet is associated with differences in DNA methylation. A study examined nutrient patterns in the Molisani cohort from Italy, and reported that higher intake of zinc and vitamin B3 is associated with higher global DNA methylation (Noro et al., 2022). Pooled results from the Women Health Initiative and the In-CHIANTI datasets show that higher fish intake is associated with lower age acceleration (Quach et al., 2017). Other studies have also focused on global diet quality. Although the scores in some specific items may vary, a diet is generally considered to be high quality when it reflects a high intake of vegetables, fruits, whole-grains, and nuts, along with a reduced consumption of added sugars, saturated fats, red processed meat, and alcohol. Epigenetic studies have shown that healthy dietary patterns are associated with decelerations in biological aging, as measured with epigenetic clocks (Kim et al., 2022; Kresovich et al., 2022).

Methodologically speaking, it is extremely difficult to examine the effects of single foods, or specific nutrients on biological outcomes, such as epigenetics. This is specially so because nutrients might interact with each other, with other lifestyle factors such as physical activity or pollution exposure, and with physiological processes such as aging or menopause. Although the previous observational studies had certain advantages to detect epigenetic changes associated with diet, such as large statistical power, clinical studies offer unique experimental settings to examine the effects of dietary patterns, macronutrients, or micronutrients in a controlled manner. In this context, in the present review we focus on clinical studies, both randomized and non-randomized. To obtain a more comprehensive view of the aging methylome, we focus on studies examining two different indexes of epigenetic methylation: i) global methylation indexes and its surrogates, such as LINE-1 methylation, and ii) epigenetic methylation clocks of biological aging.

The primary aim of our study was to determine what nutrition interventions are associated with global changes in DNA methylation as well as with more favorable outcomes in epigenetic aging. Our working hypothesis is that healthy nutrition patterns will be associated with increases in global methylation or with younger epigenetic aging trends. A secondary aim was to examine whether age and sex distribution affect the relationship between nutrition and DNA methylation.

## Methods

We performed a systematic review of the literature to explore what nutrition interventions are associated with longitudinal changes in DNA methylation. The protocol of this review was preregistered in the following address: www.osf.io/89pzj and follows the Preferred Reporting Items for Systematic Reviews and Meta-Analyses (PRISMA) guidelines (Moher et al., 2009).

**FIGURE 1:**
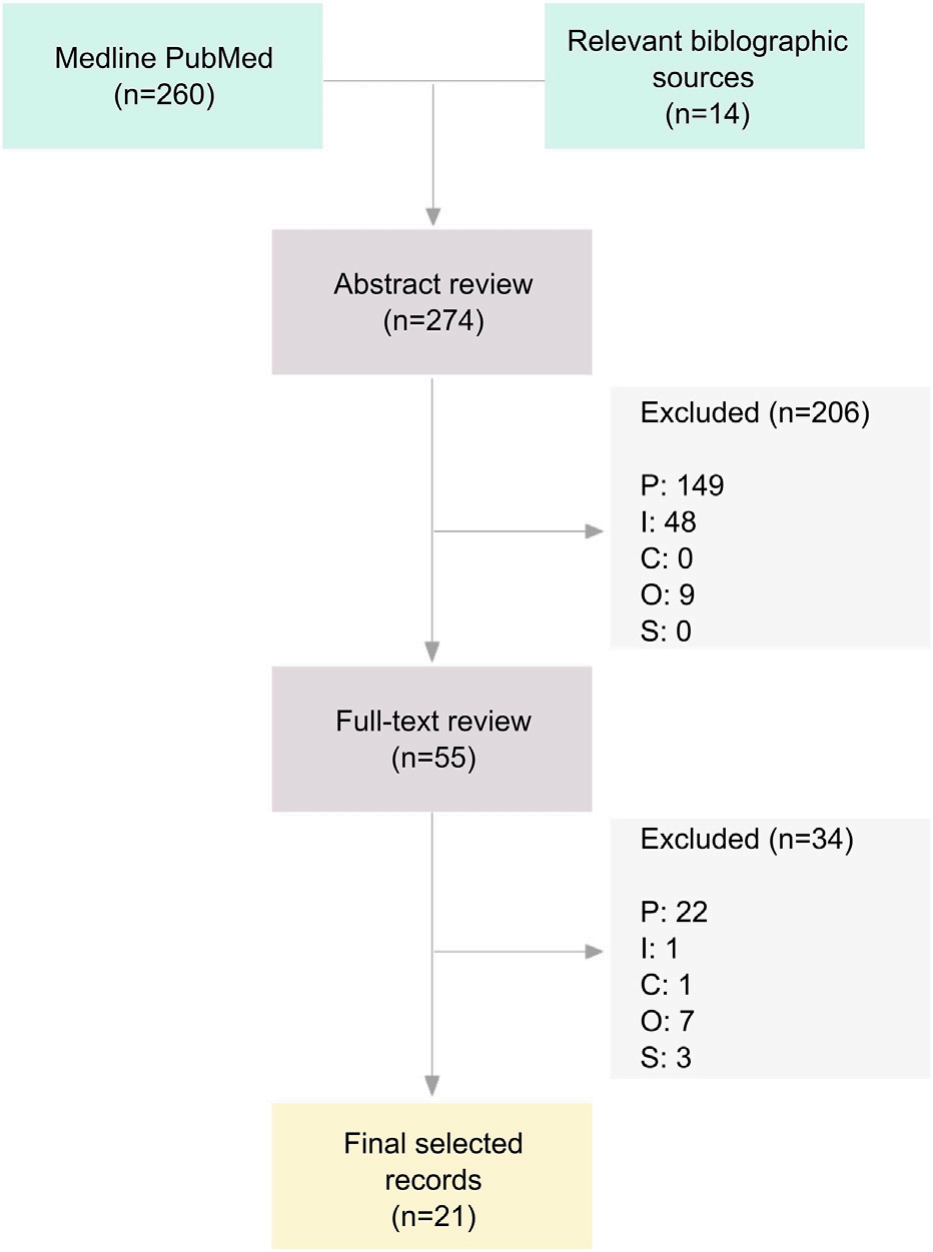
Flowchart of the screening process. *Notes:* P, Participants; I, Intervention; C, Comparison; O, Outcome; S, Study type.

We searched for peer-reviewed studies available on PubMed using the following query strings: (epigenetics or DNA methylation) and (nutrition or food or diet or lipid or carbohydrate or protein or vitamin or phytochemical or nutraceutical). Automatic filters from PubMed were applied in order to include the following types of papers: classical article, clinical studies, clinical trial, controlled clinical trial, multicenter study, observational study, RCTs. The references cited in relevant papers (e.g., systematic reviews that are close in scope (ElGendy et al., 2018) and all of the studies included) were also searched for additional studies. Two authors working in parallel performed the literature search, and disagreements were solved by consensus. We used the PICO portal platform (www.picoportal.net) to assist with the literature screening. Figure 1 shows the results of the screening process.

We included clinical studies in adults that administered a nutrition intervention and that provided measures of DNA methylation, either global methylation, proxies of global methylation (such as repetitive element methylation LINE-1), and epigenetic clocks based on methylation patterns and that were published until January 2024. The most important exclusion criteria were the following: a) prenatal studies, as well as studies on childhood or adolescence, since their results might not automatically generalize to adulthood; b) studies recruiting participants with medical comorbidities, since some of the results might be specific to the medical condition analyzed; c) animal studies; d) studies on the effects of caloric restriction or intermittent fasting, since they involve substantial changes in caloric intake and, as such, their results cannot properly inform about the effects of specific nutrients, foods, or food patterns; e) studies on alcohol intake; since we do not consider alcohol to be a nutrient; f) multimodal treatments (e.g., nutrition and meditation administered together) only if, in this last case, the study design does not allow to examine the effects of the nutrition intervention on its own (for the full list of inclusion and exclusion criteria see Table 1).

**TABLE 1:** Inclusion and exclusion criteria used in this systematic review.

	Inclusion criteria	Exclusion criteria
Population	Healthy adults aged ≥ 18 years old	Studies on childhood or adolescence, studies on medical pathologies (e.g., cancer, cardiovascular diseases, neurodegenerative disorders, *etc.*), studies on prenatal effects Animal models
Intervention	Any dietary pattern, specific food item, macronutrient, or micronutrient	Caloric restriction diets that do not provide further guidelines on a balanced nutrition pattern Effects of alcohol
Comparison	Nutritional interventions administered together with other treatments (e.g., physical activity) will be included if the design and analysis enable to approximate the effects of the nutrition intervention on its own	Multimodal treatments including a nutrition component that do not enable to isolate in a statistic test the effects of the nutrition intervention on its own
Outcome	Global methylation, proxies of global methylation (e.g., LINE-1 methylation or Alu methylation), epigenetic clocks based on methylation	Epigenetic factors other than methylation, targeted DNA methylation studies, site-specific DNA methylation
Studies	Clinical studies (with or without a control condition)	Study protocols, observational studies, reviews, meta-analysis

From the included studies, we extracted the following information: goal of the study, name of the study and country, study design, cross-over (Y/N), participants blind (Y/N), observer blind (Y/N), control condition, length of the intervention, sample size, average age, percentage of female participants, description of the nutrition intervention, description of other treatments administered, epigenetic markers, statistical analysis, control of confounders, main results. Two authors working sequentially extracted the data. If two or more studies had overlapping sample sizes and analyses, we cited both studies but extracted only the most comprehensive results.

### Deviations from the preregistered protocol

In the preregistered protocol we specified that we would exclude studies on medical pathologies. This way, we aimed at focusing on those results that are relevant to the general population, easily generalizable, and to avoid potential biases associated with some medical diseases. This criterion has been kept mostly intact, and we have excluded studies focusing on patients with cancer, cardio-vascular disorders, or neurodegeneration. However, we have relaxed our inclusion criteria to include studies recruiting participants with overweight and obesity, participants who smoke, participants with suboptimal levels of vitamin D, participants with moderately elevated values of homocysteine, and participants with high triglycerides and/or low HDL cholesterol. This decision was taken after considering that these health problems are present in a significant portion of the population, and that by including them, we can provide a real-world representation of health as a continuum spanning from optimal to suboptimal.

### Other clarifications regarding inclusion/exclusion criteria

In this review we have included a weight-loss intervention study, despite stating otherwise in the exclusion criteria (Meir et al., 2021). The inclusion of this paper is because this study targets weight loss by providing nutrition guidelines that are well aligned with the Mediterranean diet (for further details on this diet, please refer to the results section). As such, we have considered that this study provides useful insights on the link between nutrition and DNA methylation changes.

## Results

**FIGURE 2:**
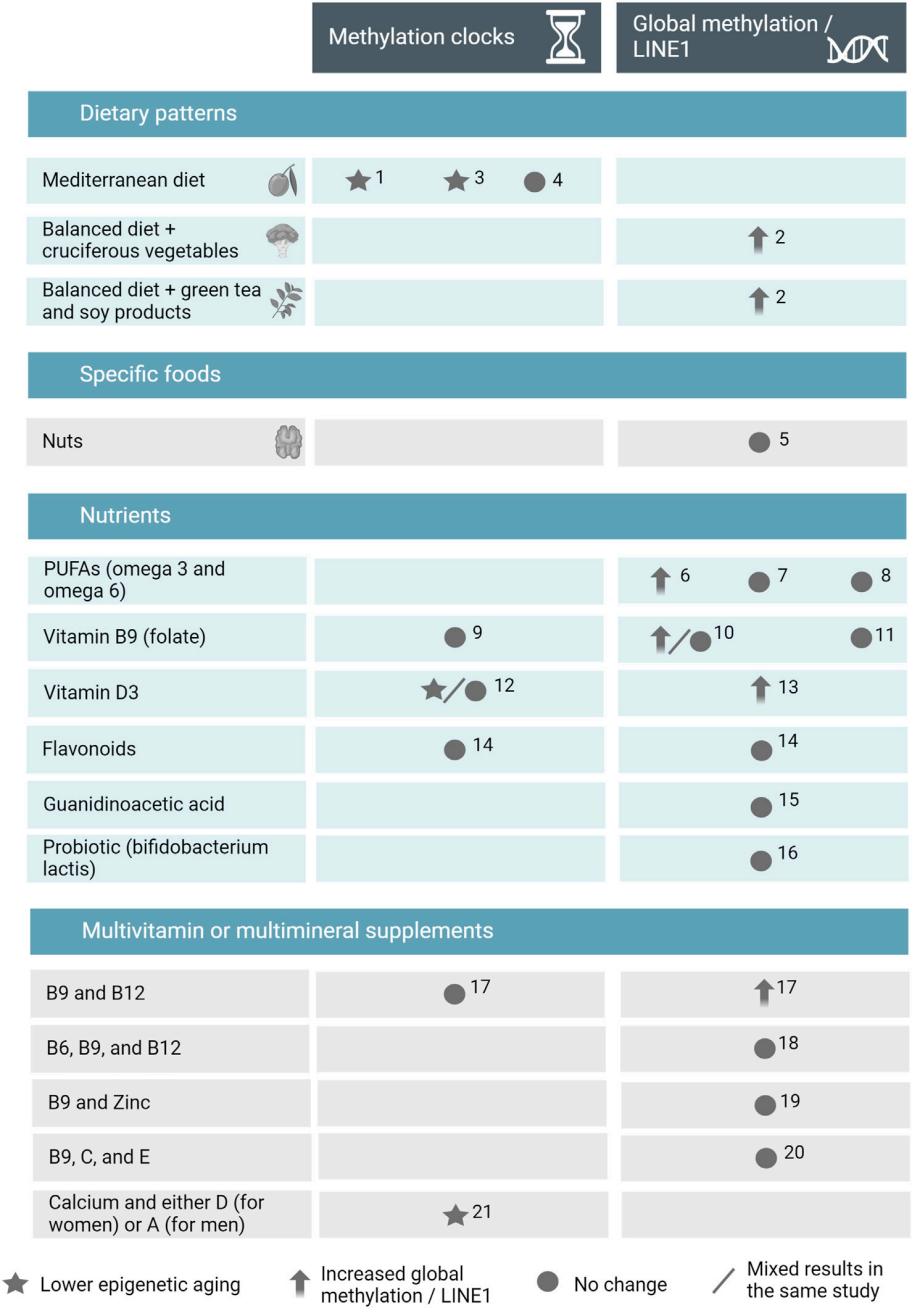
Summary of the results of the systematic review on human intervention studies in adulthood examining the effects of nutrition on DNA methylation clocks and global DNA methylation. Abbreviations: LINE1, long interspersed elements 1; PUFAs, polyunsaturated fatty acids; 1, Gensous et al., 2020; 2, Scoccianti et al., 2011; 3, Fiorito et al., 2021; 4, Meir et al., 2021; 5, Salas-Huetos et al., 2021; 6, Perfilyev et al., 2017; 7, Frankhouser et al., 2022; 8, Hunter et al., 2019; 9, Michels and Binder 2024; 10, Crider et al., 2011; 11, Jung et al., 2011; 12, Chen et al., 2019; 13, Zhu et al., 2016; 14, flavonoids study in Sae-Lee et al., 2018; 15, Ostojic et al., 2018; 16, Worthley et al., 2009; 17, vitamins B9 and B12 study in Sae-Lee et al., 2018; 18, Hübner et al., 2013; 19, Jenkins et al., 2022; 20, Chan et al., 2017; 21, Demidenko et al., 2021. Figure created with BioRender.

We identified twenty-one clinical studies that examined the effects of nutrition on either global DNA methylation, proxies of global methylation (i.e., LINE-1), or on methylation-based epigenetic clocks. Among them, thirteen studies were randomized clinical trials, while seven studies were non-randomized (Supplementary Table S1, for a summary of the results see Figure 2). Since most studies examined methylation in blood tissues, in the text we have specified if DNA methylation is examined in body tissues other than blood.

### Studies on dietary patterns

We have included four intervention studies that focused on the effects of beneficial dietary patterns on DNA methylation. The NU-AGE study (n = 120, 58% women) evaluated the impact of 1-year adherence to a Mediterranean diet on epigenetic age, in an Italian and a Polish cohort aged on average 72 years old. In this study, the Mediterranean diet intervention promoted a high intake of wholegrains, fruits, vegetables, and legumes, a moderate increase of low-fat dairy, fish, low-fat meat, eggs, nuts, and olive oil, and a limited use of alcohol, salt, and sweets. Across both cohorts, higher adherence to the Mediterranean diet was associated with lower biological age acceleration, as measured with the Horvath clock (Gensous et al., 2020).

Another study also conducted in an Italian cohort of males, with an average age of 52 years (n = 88) investigated the effects of three diets: i) an isocaloric diet with balanced fruits and vegetables, which constituted their “control” diet; ii) a regime based on their control diet that in addition included cruciferous vegetables and iii) a regime based on the control diet that included green tea and soy products. Participants were told to consume these diets for 4 weeks. The three diets were associated with small increases in LINE-1 methylation, spanning from 1% to 3% increases in methylation (Scoccianti et al., 2011).

The DAMA (Diet Physical Activity and Mammography) study (n = 219) examined a group of Italian women with an average age of 59 years old that were randomly assigned to four groups during 2 years: i) dietary intervention, in which participants were counseled to adopt a dietary pattern characterized by being rich in whole grains, fruits, vegetables, legumes, and pulses, by the use of extra-virgin olive oil, and by the reduction in red meat, alcohol intake, cheese, and sweets; ii) a physical activity intervention; iii) a combination of dietary and the physical activity interventions; and iv) no interventions. They found that the dietary intervention was associated with a reduction of epigenetic aging measured using the GrimAge clock (Fiorito et al., 2021).

Finally, the CENTRAL study (n = 120, Israel), a cohort of mostly men (92%) aged on average 49 years old, randomized participants with obesity and/or dyslipidemia to two hypocaloric diets: a low-fat diet, characterized by limiting their total fat intake to 30% of their caloric intake, and a Mediterranean diet with a low-carbohydrate pattern, characterized by being restrictive in the amount of carbohydrates and in red meat, and by being rich in vegetables, legumes, and nuts. Half of the participants in both conditions also received a physical activity intervention. The interventions lasted for 18 months. There were no effects of the nutrition intervention on epigenetic methylation age, as measured with a 240 CpGs algorithm. However, in a separate analysis examining the interaction between dietary intervention and weight loss, participants who achieved weight loss had a significantly lower increase in epigenetic age after the intervention (0.6 years) relative to participants who did not lose weight (1.1 years) (Meir et al., 2021).

### Studies on the effects of specific foods

One study has focused on the epigenetic effects of specific foods.

The FERTINUTS study, examined Spanish men aged on average 25 years old (n = 72), and investigated the effects of nuts in global methylation changes in sperm after a 14-weeks intervention study. The study reported a lack of differences in global methylation changes between participants assigned to the nut intervention and participants who did not include nuts in their diet (Salas-Huetos et al., 2021).

### Studies on specific macro- or micro-nutrients

We identified ten clinical studies that focused on the effects of specific macro- or micro-nutrients on global DNA methylation or on epigenetic clocks.

Regarding the effects of lipids, a study performed a secondary analysis of a larger randomized clinical trial performed in U.S. women, in which they selected a small subset of participants (n = 10, average age 51 years old), which showed good responses to omega-3 polyunsaturated fatty acids supplementation, taken for 6 months. This subset of participants was further analyzed for epigenetic changes. The authors reported no change in global DNA methylation after treatment with omega-3 polyunsaturated fatty acids (Frankhouser et al., 2022). Similarly, a study conducted in men (n = 8, average age 40 years old) performed in the UK used a cross-over design and administered either omega-3 fatty polyunsaturated fatty acids or a supplement of extra virgin olive oil for 4 weeks. None of the fatty acid supplements altered global methylation (Hunter et al., 2019). A third trial, The LIPOGAIN study, conducted in Sweden (n = 31, average age 27 years old, 36% women) evaluated methylation changes in the adipose tissue after a 7-weeks intervention study, in which participants were randomized to consume muffins with either added palm oil (rich in saturated fats) or added sunflower oil (rich in omega-6 polyunsaturated fatty acids). Both oils induced higher mean methylation in the adipose tissue (Perfilyev et al., 2017).

The isolated effects of folic acid supplementation have been tested in three studies. A randomized cross-over study performed on Chinese women tested the effects of folic acid over 6 months in global methylation extracted from coagulated (n = 135, average age 30 years old) and uncoagulated (n = 76 subsample) blood analysis. While global methylation remained unchanged when extracted from uncoagulated blood, there was a reduction in global methylation after folic acid supplementation, when this measure was obtained from coagulated blood (Crider et al., 2011). The FACIT trial (Folic Acid and Carotid Intima Media Thickness, the Netherlands) is a randomized double-blinded trial that recruited participants with moderately elevated homocysteine (n = 216, average age 61 years old, 45% women) and tested the effects of folic acid supplementation for 3 years on global DNA methylation. There was no difference in DNA methylation between placebo and treatment with folic acid, and no different effect was observed when stratifying the sample by age or by sex (Jung et al., 2011). Finally, a randomized clinical trial conducted in Germany recruited male participants (n = 16, average age 39 years old) and tested the effect of supplementation of 400 μg/d vs. 800 μg/d of folic acid for 8 weeks on different methylation clocks (i.e., Horvath’s, Hannum’s, PhenoAge, GrimAge, GrimAge2, and DunedinPACE). None of the folic acid supplementation regimes elicited changes on the methylation clocks (Michels and Binder, 2024).

Two studies examined the effects of vitamin D supplementation. A randomized clinical trial on overweight African-American participants with suboptimal levels of vitamin D (n = 51, average age 26 years old, 84% women) administered a supplement of D for 16 weeks using four conditions: placebo, 600 lU/d, 2000 lU/d, and 4,000 lU/d of vitamin D. They found that, in the intervention groups of 2000 and 4000 IU/d, epigenetic methylation age measured with the Horvath clock was decreased by 1.83 and 1.62 years compared to baseline. However, when they measured epigenetic age using another clock (Hannum’s) no results were found (Chen et al., 2019). Using the same study design and sample, another paper already showed that increases in global DNA methylation associated with vitamin D supplementation were dose-dependent, that is, increases in global methylation were the highest after 4000 IU/d of vitamin D, followed by 2000 IU/d, and followed by 600 IU/d, relative to placebo (Zhu et al., 2016).

Regarding flavonoids, one study looked at DNA methylation on 13 participants (average age 22 years old, 57% women) who, as part of a larger study, had been previously assigned to receive supplementation with monomeric and oligomeric flavonols derived from grape seeds for 8 weeks. They found no differences before and after supplementation neither in global methylation nor in the epigenetic age measured with the Horvath clock (Sae-Lee et al., 2018). Part of these results (global DNA methylation) were also reported in another overlapping publication (Milenkovic et al., 2014).

One study (n = 14) administered a supplement of guanidinoacetic acid, a precursor of creatine, for 12 weeks to young adults. They found no differences in global DNA methylation (Ostojic et al., 2018).

Finally, a randomized clinical trial (n = 20, average age 60 years old, 35% women) examined whether a 4-week administration of either a probiotic (*bifidobacterium lactis*), a prebiotic (high amylose maize starch), or a synbiotic (administration of the prebiotic and the probiotic together) changed LINE-1 methylation in the rectal mucosa. After the dietary interventions, mean LINE-1 methylation remained unchanged (Worthley et al., 2009).

### Studies examining the effects of multivitamin or multicompound supplementation

Five studies have examined the effects of multivitamin or multicompound supplements on global methylation markers.

Among them, two studies have focused on sperm DNA methylation. A large randomized double-blind placebo-controlled trial named the FAZST (Folic Acid and Zinc Supplementation), performed in the U.S. examined the effects of a 6-month supplementation using folic acid and zinc on the DNA methylation sperm of men (n = 1,470, average age: 33 years old). They reported no effects of zinc and folic acid supplementation on global DNA methylation (Jenkins et al., 2022). In another study conducted in the U.S., men (n = 8, 18–32 years old) were randomized to receive a multivitamin supplement (vitamin C, vitamin E, and folic acid) or placebo for 3 months. There were no significant changes in sperm DNA methylation after the multivitamin intervention (Chan et al., 2017).

Another study (n = 42, average age 63 years old, 33.3% women) administered Rejuvant^®^, a compound with two formulations: one for women, containing calcium-alpha-ketoglutarate and vitamin D, and one for men, containing calcium-alpha-ketoglutarate and vitamin A. They calculated epigenetic methylation age based on a proprietary algorithm which showed a moderate correlation with chronological age in the same sample (r = 0.77). They found that after 4–10 months of treatment with Rejuvant^®^, epigenetic age was decreased by approximately 8 years, and that the effects were similar for men and for women (Demidenko et al., 2021).

Two studies have examined the effect of combined B-complex vitamins. A randomized double-blind placebo-controlled trial (n = 44, 65–75 years old, 57% women) tested the effects of a folic acid and vitamin B12 supplement taken during 2 years on global methylation and on epigenetic age, as evaluated with the Horvath clock. While the analyses of epigenetic age had no significant results, the administration of folic acid and vitamin B12 was shown to increase global DNA methylation (Sae-Lee et al., 2018). Another randomized double-blind trial (n = 50, average age 68 years old, 71% women) studied the effects of a 1-year administration of two food supplements: a control one, consisting of vitamin D and calcium, and one that contained B-vitamins (folic acid, vitamin B6, and vitamin B12) along with vitamin D and calcium. LINE-1 methylation remained unchanged before and after the supplementation, as analyzed with a paired-samples t-test (Hübner et al., 2013). Of note, this same analysis was recycled by the same authors in a posterior publication with a different statistical test (linear regression). As in their first analysis, mean LINE-1 methylation remained unchanged after supplementation (Pusceddu et al., 2016).

## Discussion

In this review, we have evaluated whether nutrition interventions can change global DNA methylation patterns. With regards to the effect of dietary patterns, we identified four independent studies that examined the effects of a “healthy” diet, characterized by being rich in vegetables, fruits, legumes, and nuts, and by aiming to reduce the intake of red meat, added sugars, and alcohol. Three of the studies concluded that this healthy dietary pattern is associated with DNA methylation changes. Among them, two studies lower accelerations in epigenetic aging (Gensous et al., 2020; Fiorito et al., 2021), and one of them showed higher LINE-1 methylation measures (Scoccianti et al., 2011). An additional study reported no significant effects on DNA methylation (Meir et al., 2021). Although the evidence is still scarce to draw firm conclusions, results begin to point in the direction that a beneficial nutritional regime might have positive effects on DNA methylation. Healthy dietary patterns provide good sources of vitamins, such as vitamins A, C, E, or B-complex vitamins, minerals such as magnesium, selenium, and zinc, and other nutrients such as polyunsaturated fatty acids, flavonoids, and isoflavones. These nutrients constitute important components of the so-called *anti-inflammatory diet*, a dietary pattern associated with lower levels of circulating proinflammatory cytokines (Shivappa et al., 2014). Adherence to anti-inflammatory dietary patterns has been associated with small reductions in the risk of developing inflammatory conditions such as rheumatoid arthritis (Hu et al., 2017; Gill et al., 2022) as well as with improvements of chronic pain in such conditions (Schönenberger et al., 2021). We interpret the findings of our systematic review as preliminary evidence suggesting that nutrition interventions that provide a balanced intake of different macro- and micro-nutrients might be particularly well-equipped to induce long-term changes in DNA stability and biological aging mechanisms. It has been suggested that particular nutrients, such as B-vitamins and choline, induce changes in DNA methylation by acting on the one-carbon metabolism pathway, a mechanism that provides methyl groups for reactions in methylation (Choi and Friso, 2010; Amenyah et al., 2020). The intake of dietary polyphenols, such as flavonols or isoflavones, can change the composition of the gut microbiome, with potential implications for the promotion of gastrointestinal health. Moreover, polyphenol metabolites originating from the gut microbiome might act on epigenetic mechanisms by reducing inflammatory biomarkers and regulating oxidative stress (Scott et al., 2022). In fact, polyphenols seem to influence methylation in genes that are critical for cancer (Choi and Friso, 2010).

Studies on the effects of single foods, single nutrients, or multivitamin/multimineral supplements were generally scarce and produced conflictive findings. For instance, both in the case of folic acid and polyunsaturated fatty acids, independent studies yield nonconvergent results. Sometimes, in the same paper, different methods yielded different results, which cautions against an optimistic overinterpretation of the positive findings (e.g., the examination of coagulated vs. uncoagulated blood in Crider et al., 2011).

We did not find age or sex-related trends in the association between nutrition and methylation changes. At the same time, the relations between nutrients and methylation changes highlighted here might differ considerably at early developmental stages, which were not covered in this review. For example, although we found no solid evidence that folic acid has the capacity to modify the global methylation in adulthood, B-complex vitamins might be fundamental during the early embryonic period, since alterations in epigenetic reprogramming might explain neural tube defects caused by folate deficits in early embryonic stages (Choi and Friso, 2010).

In our review, no study on the epigenetic effects of dietary polysaccharides met inclusion criteria. Dietary polysaccharides are complex carbohydrates composed of long chains of sugar units that are present in foods such as whole grains, vegetables, and fruits. Cell studies and research in rodents have shown that polysaccharides from natural sources such as resistant starch (RS4) and astragalus can induce epigenetic changes such as histone modification and DNA methylation (Guo et al., 2023; Liu et al., 2023). Further research with human clinical trials is needed to confirm these epigenetic effects observed in these pre-clinical investigations.

We acknowledge several limitations in our review. First, all the studies were conducted in participants living in Europe and North America and Australia, and our results do not automatically generalize to populations in other parts of the world. Second, the validity of our conclusions is constrained by the quality of the original articles, and we have included some non-randomized studies and trials performed in a very small number of participants, which increases the risk of bias. Third, while global increases in DNA methylation or in its surrogates are interpreted in the original papers as a favorable epigenetic outcome, it is known that age also causes increases in DNA methylation at the CpG sites (Seale et al., 2022). For this reason, the interpretation of results on higher global DNA methylation as positive can be oversimplistic and should ideally be confirmed with other global measures of the DNA methylation profile, such as epigenetic clocks. Fourth, it is still a matter of debate whether improvements in epigenetic age reflect ameliorations in biological aging. For instance, a study on chronic kidney disease found that a group of patients that underwent kidney transplantation had significant reductions in epigenetic age acceleration 1 year after transplantation. While this result is very encouraging, there is a caveat: the authors found no significant differences in epigenetic age acceleration between a control group of healthy individuals at baseline and the group patients with kidney transplantation 1 year after their surgery. This raises doubts about the validity of epigenetic clocks, especially since patients with kidney transplantation have a reduced life expectancy relative to controls, as it was acknowledged by the same authors (Neytchev et al., 2024). The inclusion of different epigenetic clocks might partially overcome this issue since some epigenetic clocks have been specially designed to be more sensitive to the occurrence of adverse health effects. Despite this, only a few of the studies included here included different epigenetic clocks in their analyses (i.e., Chen et al., 2019; Michels and Binder, 2024).

In conclusion, we have examined what specific nutrition variables are able to induce DNA methylation changes in community-dwelling adults. Evidence from clinical studies is still scarce, and a higher number of well-powered randomized clinical studies are necessary to conclude upon the effects of nutrition on methylation and biological aging. Initial evidence starts to suggest the possibility of positive epigenetic outcomes related to healthy nutrition patterns, but these results should be regarded as preliminary. To draw definitive conclusions, we will need to wait for the results of robust clinical trials.
